# Flexural Behavior of Steel-FRP Composite Bars (SFCB)-Reinforced Concrete Beams: FEA Incorporating Bond-Slip Effects

**DOI:** 10.3390/ma18225226

**Published:** 2025-11-18

**Authors:** Chaohao Bi, Shuo Xu, Yu Ling, Yicong Zhong, Linbo Hong, Yongjian Cai

**Affiliations:** 1 Guangzhou Power Supply Bureau, Guangdong Power Grid Co., Ltd., China Southern Power Grid Co., Ltd., Guangzhou 510665, China; 2 School of Intelligent Transportation and Engineering, Guangzhou Maritime University, Guangzhou 510725, China; 3 School of Civil and Transportation Engineering, Guangdong University of Technology, Guangzhou 510006, China

**Keywords:** steel-FRP composite bars (SFCBs), bond-slip effect, flexural behavior, finite element analysis, FRP thickness, ductility index, failure mode

## Abstract

To overcome the corrosion issues of conventional steel reinforcement and the brittleness of fiber-reinforced polymer (FRP) materials, steel-FRP composite bars (SFCBs) offer an innovative solution by combining the ductility of steel with the high strength and corrosion resistance of FRP. However, existing research primarily focuses on experimental investigations, with insufficient numerical simulations of SFCB-reinforced concrete beams, particularly regarding bond-slip effects at the SFCB-concrete interface—a critical mechanism governing composite action and structural performance. This study develops a finite element (FE) model incorporating SFCB-concrete bond-slip effects to analyze the influence of outer FRP layer thickness (0, 3, 5, and 7 mm) on the flexural performance of concrete beams. The FE model demonstrates good predictive accuracy, with errors in ultimate capacity and mid-span displacement within 7% and 8%, respectively. Both cracking and yield loads increase with FRP thickness, while the ultimate load peaks at 5 mm. At 7 mm, concrete crushing occurs before the SFCB reaches its ultimate strength. The ductility index decreases with greater FRP thickness due to increased elastic energy without enhanced plastic energy (fixed steel core area), thereby reducing overall ductility. These findings provide a theoretical basis for optimizing SFCB-reinforced concrete structural design.

## 1. Introduction

Reinforced concrete structures are extensively utilized in modern buildings and bridges due to excellent mechanical properties and cost-effectiveness. However, they face durability challenges in corrosive environments where corrosion of steel degrades the bond between steel and concrete [1,2,3]. Chloride ingress or carbonation can destroy the passive film on steel reinforcement. This destruction initiates corrosion. The corrosion of steel reinforcement significantly degrades beam performance through two primary mechanisms. First, corrosion reduces the effective cross-sectional area of the steel bar. This reduction directly decreases its yield strength and ductility. Second, corrosion products have a larger volume than the original steel. This expansion generates substantial hoop tensile stresses within the concrete. Consequently, these stresses cause the concrete cover to spall and severely impair the bond between the steel and concrete [4]. The degradation of this bond-slip effect alters the load-bearing mechanism of the beam. It leads to increased crack widths and greater deflections. Ultimately, this results in a significant reduction in the beam’s load-carrying capacity. Furthermore, the failure mode shifts from a ductile manner to a more brittle one [5].

Fiber-reinforced polymer bars (FRP), recognized for superior strength-to-weight ratios and fatigue resistance, offer an alternative to steel reinforcement for enhanced durability [6,7,8,9]. Nevertheless, linear-elastic behavior of FRP induces brittle failure with limited failure warnings in FRP-reinforced concrete structures [10,11,12,13,14]. Steel-FRP composite bars (SFCBs) address these limitations by synergizing the ductility of steel with the high strength and corrosion resistance of FRP [15,16]. Compared to pure FRP bars [17,18], SFCBs exhibit higher initial elastic modulus and cost efficiency. Relative to traditional steel, they provide superior corrosion resistance and customizable post-yield stiffness [19,20]. Mechanically, SFCBs show bilinear stress–strain behavior with stable post-yield stiffness, minimal residual deformation, and repairability. This enables damage-controllable designs that meet repairability standards after moderate earthquakes [21,22,23].

In recent years, significant progress has been made in the study of SFCB-reinforced concrete (SFCB-RC) beams [24,25,26,27]. SFCBs improve flexural strength and stiffness and exhibit a distinct secondary stiffness. After yielding of the steel core, the surrounding fibers continue to provide a stable increase in load-carrying capacity, leading to a hardening feature in the load-deformation response [10,28,29]. Compared with FRP bars, at the same reinforcement ratio, SFCB can change the failure mode of beams from brittle fracture to ductile failure, effectively avoiding sudden collapse [30]. The fiber volume fraction is a key factor affecting flexural performance: more fiber improves crack control and stress transfer, delaying diagonal crack growth. A higher steel core ratio increases beam stiffness, while concrete strength has a minor influence [31]. Ge et al. [21] investigated the flexural behavior of concrete beams reinforced with non-prestressed SFCB and prestressed FRP bars and identified two bending failure modes in FRP-prestressed concrete beams. Bai et al. [25] examined the static flexural behavior of composite beams reinforced with SFCB and engineered cementitious composites (SFCB-RC/EC) and demonstrated that, compared with conventional SFCB-RC beams, the corresponding SFCB-RC/EC specimens exhibited a 38.3% increase in load-carrying capacity and a 42.8% improvement in energy ductility index, significantly enhancing beam deformability. A simplified analytical model for predicting the flexural capacity of SFCB-RC/EC beams was also proposed. Overall, by integrating the ductility of steel with the high strength and corrosion resistance of fibers, SFCB overcomes the durability limitations of traditional reinforced concrete and the brittle failure drawback of FRP reinforcement.

However, research on SFCB-RC beams remains predominantly experimental, with limited numerical simulations—particularly regarding finite element models incorporating bond-slip effects [32]. Finite element analysis is an efficient tool that reduces the cost and time of physical tests while providing detailed mechanical insights, such as concrete damage and reinforcement stress distribution [33]. In traditional finite element analysis of steel-reinforced concrete beams, perfect bonding between steel and concrete is commonly assumed, neglecting relative slip [34]. However, extensive experimental studies demonstrate that this simplification causes significant deviations from actual behavior, especially under high loads or complex stress states [35]. For example, comparative analyses using DIANA software by Xu et al. [36] revealed that finite element models considering bond-slip effects predict cracking load, deflection, and crack distribution more accurately, whereas models ignoring slip substantially underestimate structural nonlinearity.

Unlike conventional steel rebars, FRP bars exhibit more pronounced brittle debonding in bond-slip behavior with concrete [37,38,39]. The steel-concrete bond is dominated by mechanical interlock, whereas chemical adhesion and friction contribute minimally and are significant only during initial slip [40]. In contrast, the bond mechanism between FRP bars and concrete comprises chemical adhesion, friction, and mechanical interlock [41]. As a novel composite reinforcement, the interfacial bond mechanism of SFCBs with concrete involves greater complexity [42]. Consequently, establishing an accurate constitutive model reflecting SFCB-concrete bond-slip behavior is essential for precise simulation of the mechanical performance in SFCB-RC beams.

Based on this context, this study focuses on SFCB-RC beams. A finite element model incorporating bond-slip effects is established using ABAQUS to investigate flexural failure modes of SFCB-steel concrete beams and analyze the influence of outer FRP layer thickness on their flexural performance. The results provide a theoretical basis for designing SFCB concrete components and promote their engineering applications.

## 2. Finite Element Model

The core objective of this study is to develop and validate a high-fidelity three-dimensional finite element model. This model accurately simulates the flexural behavior of SFCB-RC beams. A specific focus is placed on incorporating the bond-slip effect at the SFCB-concrete interface. The overall workflow is outlined as follows. (1) Geometric Modeling and Discretization: A three-dimensional model of the beam specimen was created based on the experimental dimensions. Appropriate element types were selected for different components, including concrete, steel bars, and interfaces. (2) Material Constitutive Modeling: Nonlinear material models were precisely defined for concrete, steel, GFRP, and the SFCB. These models capture the unique stress–strain characteristics of each material. (3) Interface Characterization: The critical bond-slip behavior between the tensile reinforcement and the surrounding concrete was simulated. A cohesive surface interaction model, following a traction-separation law, was employed for this purpose. (4) Model Validation: The developed FE model was validated by comparing its predictions with experimental data from control specimens. The comparisons focused on the load–displacement response and failure modes. (5) Parametric Analysis: The validated model was utilized to conduct a systematic parametric study. The primary variable investigated was the thickness of the outer FRP layer. Thicknesses of 3 mm, 5 mm, and 7 mm were considered to evaluate their influence on flexural performance. A detailed description is provided in the following sections.

**Figure 1:**
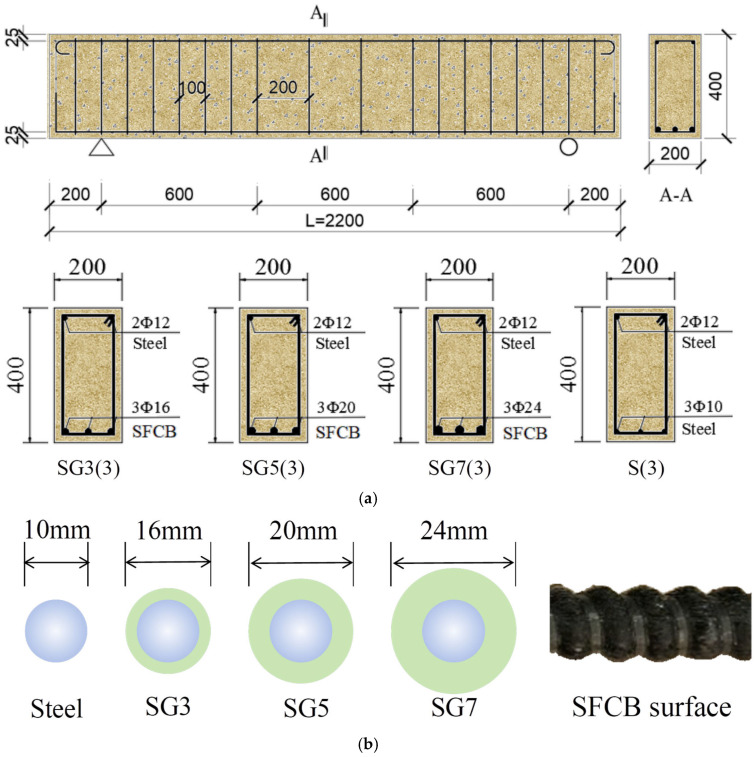
Schematic of specimens and reinforcement details: (**a**) Beam specimen dimensions and reinforcement layout; (**b**) Reinforcement bar dimensions.

**Figure 2:**
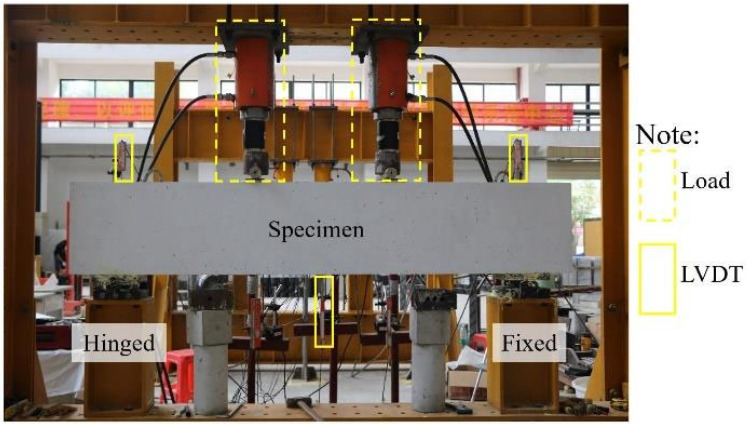
Tests on SFCB-RC beams.

As an illustrative example, the SFCB-RC beam had a length of 2200 mm and a cross-sectional dimension of 200 mm × 400 mm, as shown in Figure 1a. The concrete strength grade was C30. The specimen dimensions and reinforcement layout were determined with reference to the flexural design method of conventional RC beams, in which the RC beam section was designed as a balanced-reinforcement section [43,44]. All other specimens were fabricated with the same cross-sectional dimensions. The longitudinal reinforcement details are shown in Figure 1b. For RC beams, the longitudinal bars were 10 mm-diameter steel rebars. For SFCB beams, the longitudinal reinforcement consisted of SFCBs with three different outer fiber thicknesses (3 mm, 5 mm, and 7 mm), while the steel core diameter of all SFCBs was fixed at 10 mm. Double-leg stirrups with a diameter of 10 mm were used, and the compression reinforcement consisted of 12 mm-diameter steel rebars. The stirrup spacing was 100 mm in the confined regions and 200 mm in the unconfined regions. All longitudinal rebars were bent into 180° hooks at both ends. The concrete cover thickness was 25 mm. For comparison with the finite element results, experiments on corresponding specimens were conducted, as described in Figure 2.

The detailed parameters of the beam specimens are summarized in Table 1. Here, *A_s_* and *A_f_* denote the cross-sectional areas of the steel bars and GFRP bars, respectively. *ρ_s_*, *ρ_f_*, *ρ_h_* and *ρ_hE_* represent the steel reinforcement ratio (*ρ_s_* = *A_s_*/*bh*_0_), the GFRP reinforcement ratio (*ρ_f_* = *A_f_*/*bh*_0_), the practical reinforcement ratio (*ρ_h_* = *ρ_s_* + *ρ_f_*), and the nominal reinforcement ratio based on elastic modulus conversion (*ρ_hE_* = *ρ_s_* + *ρ_f_E_f_*/*E_s_*). Additionally, *b* and *h*_0_ denote the beam width and the effective depth of the section, while *E_f_* and *E_s_* are the elastic modulus of the GFRP bars and steel bars, respectively. For clarity, the specimens were designated according to the type of longitudinal reinforcement, the fiber thickness of the SFCB, and the number of longitudinal bars. For example, “SG5(3)” denotes a beam with three longitudinal SFCBs, each having a fiber thickness of 5 mm.

### 2.1. Materials and Interface Properties

The finite element (FE) model for the four-point bending test of SFCB/steel reinforced concrete beams consists mainly of concrete, reinforcement bars (including steel bars, GFRP and SFCBs), and the bond interface between the reinforcement and concrete. Therefore, selecting appropriate material models and accurately representing the mechanical behavior of the bond interface are essential for developing the FE model.

#### 2.1.1. Concrete

The concrete was designed for the C30 strength grade. The constituent materials included Ordinary Portland Cement (P.O 42.5R), Zone II medium river sand as the fine aggregate, and 5–20 mm continuously graded granite crushed stone (apparent density: 2650 kg/m^3^; crushing index: 8.5%) as the coarse aggregate. Tap water was used for mixing. The mix proportion by mass per cubic meter was designed as follows: cement 315.38 kg, river sand 714.25 kg, crushed stone 1165.36 kg, and water 205 kg, yielding a sand ratio of 38% and a water-cement ratio of 0.65. The compressive strength was tested on 150 mm cubes after 28 days of standard curing, using a Matest 400t machine (Matest, Arcore, Italy). The average value from three specimens is reported. The elastic modulus was determined using 150 × 300 mm prisms. The 28-day cube compressive strength, prism compressive strength, and elastic modulus of the river sand concrete were 26.71 MPa, 26.00 MPa, and 27.92 GPa, respectively.

**Figure 3:**
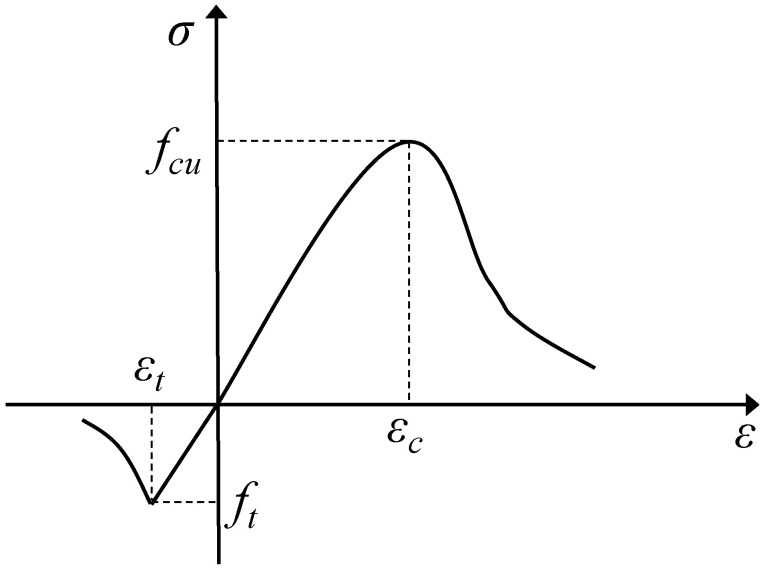
Concrete uniaxial stress–strain relationship.

The mechanical behavior of concrete under uniaxial compression and uniaxial tension differs significantly, with a commonly accepted strength ratio of approximately 10:1. Based on previous studies by both domestic and international researchers, the concrete damage plasticity model [45] was adopted in this study. The uniaxial stress–strain relationship is shown in Figure 3.

The elastic modulus of concrete was calculated according to the standard [46] (Equation (1)), with Poisson’s ratio taken as 0.2.

(1) $$E_{c}=\frac{10^{5}}{2.2+\frac{34.7}{f_{cu,k}}}$$

In the equations, *E_c_* and *f_cu_*_,*k*_ represent the standard values of the concrete elastic modulus and compressive strength. The uniaxial tensile behavior of concrete is expressed as:

(2) $$\sigma =(1-d_{t})E_{c}\varepsilon$$

(3) $$d_{t}=\left\{\begin{array}{c} 1-\rho_{t}(1.2-0.2x^{5}),\;\;\;\;\;x\leq 1\\ 1-\frac{\rho_{t}}{\alpha_{t}{\left(x-1\right)}^{1.7}+x},\;\;x>1 \end{array}\right.$$

(4) $$x=\frac{\varepsilon }{\varepsilon_{t}}$$

(5) $$\rho_{t}=\frac{f_{t}}{E_{c}\varepsilon_{t}}$$

where *α_t_*, *f_t_*, *ε_t_* and *d_t_* denote the shape parameters of the descending branch, tensile strength, peak tensile strain, and damage evolution parameter. The uniaxial compressive behavior is expressed as:

(6) $$\sigma =(1-d_{c})E_{c}\varepsilon$$

(7) $$d_{c}=\left\{\begin{array}{c} 1-\frac{\rho_{c}n}{n-1+x^{n}},\;\;x\leq 1\\ 1-\frac{\rho_{c}}{\alpha_{c}{\left(x-1\right)}^{2}+x},\;\;x>1 \end{array}\right.$$

(8) $$\rho_{c}=\frac{f_{c}}{E_{c}\varepsilon_{c}}$$

(9) $$n=\frac{E_{c}\varepsilon }{E_{c}\varepsilon_{c}-f_{c}}$$

(10) $$x=\frac{\varepsilon }{\varepsilon_{c}}$$

where *α_c_*, *f_c_*, *ε_c_* and *d_c_* denote the shape parameters of the descending branch, compressive strength, peak compressive strain, and damage evolution parameter. Based on relevant literature [45], the recommended values of the damage plasticity parameters used for concrete in the FE model are listed in Table 2.

#### 2.1.2. Reinforcement Bars

**Figure 4:**
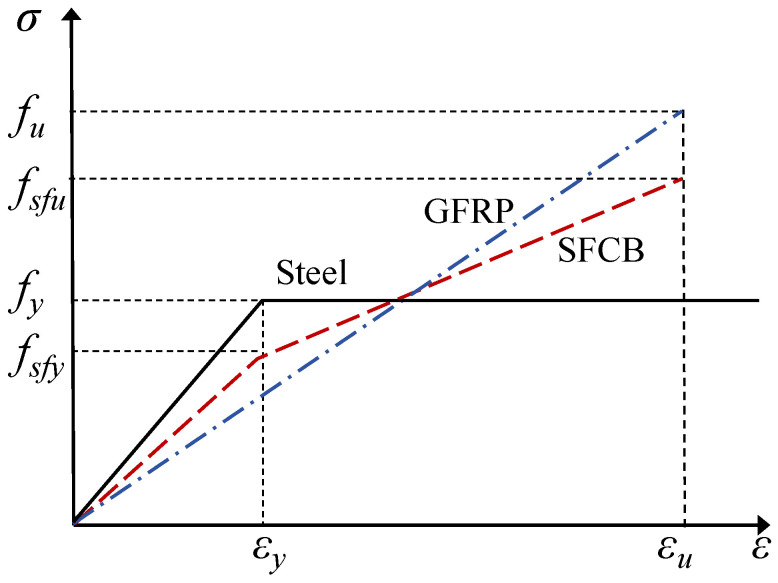
Uniaxial tensile stress–strain relationships of steel bars, GFRP bars and SFCB.

The reinforcement bars include HRB400 hot-rolled ribbed steel bars, GFRP bars, and SFCBs. The outer FRP layer of SFCBs is made of GFRP. Three specimens were prepared for each type of reinforcement to determine their mechanical properties. The tensile properties of the reinforcement are listed in Table 3. In the table, S and SG represent steel bars and SFCBs, respectively, *t_f_* and *D* denote the thickness of the GFRP layer and bar diameter; *E_I_* and *E_II_* represent the modulus of elasticity and the slope of the strain-hardening segment; *f_y_* and *f_sfy_* are the yield strengths of steel bars and SFCBs; *f_fu_*, *f_su_* and *f_sfu_* are the ultimate tensile strengths of GFRP, steel bars, and SFCBs. The stress–strain relationships of all reinforcements are shown in Figure 4.

Steel bars are assumed to be homogeneous and isotropic. For simplification, an ideal elastic-plastic model was adopted. The stress–strain curve is linear elastic up to the yield strength *f_y_*, and stress remains constant beyond *f_y_*, with the constitutive equation:

(11) $$\;\sigma =\left\{\begin{array}{c} Eɛ\;ɛ\leq {ɛ}_{y}\\ f_{y}\;ɛ>{ɛ}_{y} \end{array}\right.$$

where *E*, *σ*, *ε*, *ε_y_*, and *f_y_* are the elastic modulus, stress, strain, yield strain, and yield stress of steel bars, respectively.

For GFRP bars, due to their linear elastic behavior, a linear elastic model was adopted under uniaxial tension, with the constitutive relationship:

(12) $$\sigma_{f}=E_{f}\varepsilon_{f}$$

where *σ_f_* and *ε_f_* are the tensile stress and strain of the FRP bars.

For SFCBs, uniaxial tensile tests show that their stress–strain response can be approximated by a bilinear ideal elastic-plastic model, as shown in Figure 3, with constitutive equations given by Equations (13) and (14).

(13) $$\;\sigma =\left\{\begin{array}{c} E_{I}\varepsilon \\ E_{I}\varepsilon_{y}+E_{\mathrm{II}}(\varepsilon -\varepsilon_{y}) \end{array}\;\;\;\right.\begin{array}{c} \;0\;\leq \;\varepsilon \leq {\;\varepsilon }_{y}\\ \varepsilon_{y}\;\leq \;\varepsilon \;\leq {\;\varepsilon }_{u} \end{array}$$

(14) $$\begin{array}{c} E_{I}=(E_{s}A_{s}+E_{f}A_{f})/A\\ E_{\mathrm{II}}={(E}_{f}A_{f})/A \end{array}$$

Here, *ε_y_* and *ε_u_* are the yield and ultimate strains of SFCBs; *E_s_* and *A_s_* are the elastic modulus and cross-sectional area of the steel core; *E_f_* and *A_f_* are the elastic modulus and cross-sectional area of the FRP outer layer; *A* is the total cross-sectional area of the SFCB.

#### 2.1.3. SFCB–Concrete Interface

**Figure 5:**
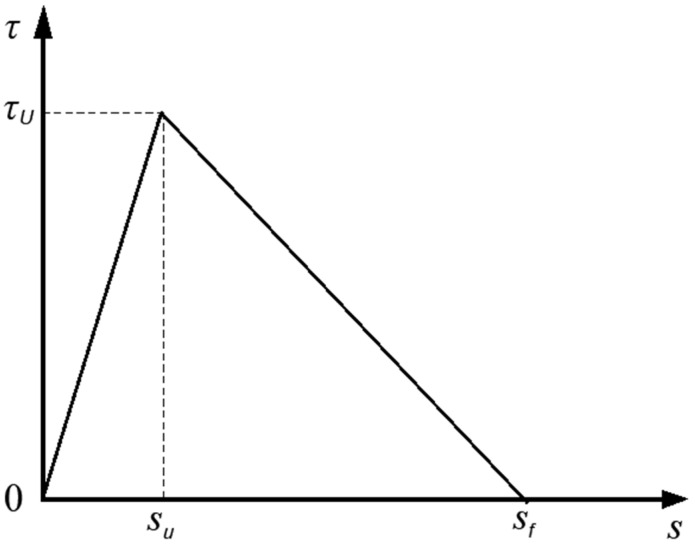
Double-line bonding-slip model.

The bond performance of the SFCB-concrete interface is fundamental for the composite action and resistance to external loads. Therefore, accurately representing the bond-slip behavior is critical for numerical simulation of pull-out tests. In this study, a bilinear bond-slip model (Model I) [47] was adopted, as shown in Figure 5 and Equation (15):

(15) $$\tau =\left\{\begin{array}{c} \tau_{u}\frac{s}{s_{u}},\;\;s\leq s_{u}\\ \tau_{u}\frac{s_{f}-s}{s_{f}-s_{u}},\;\;s_{u}<s\leq s_{f}\\ \begin{array}{c} 0,\;\;s>s_{f} \end{array} \end{array}\right.$$

where *τ_u_*, *s_u_*, and *s_f_* are the maximum bond stress, slip at peak stress, and final slip, respectively. Owing to the similar surface characteristics between SFCBs and FRP bars, as well as the analogous bond failure mechanisms of SFCB-concrete and FRP-concrete interfaces, Huang et al. [48] recommended the bond strength prediction formula provided in ACI 440.1R-15 to estimate the interfacial bond strength of SFCB-concrete (Equation (16)). Furthermore, the *s_u_* values for the specimens range from 2.8 to 5.2. According to Lin et al. [49], *s_u_* under pull-out failure is controlled by the rib spacing (*r_s_*). Therefore, the average value of *s_u_*/*r_s_* is adopted in this study, yielding the relationship *s_u_* = 0.36 *r_s_*, where *r_s_* denotes the rib spacing of the SFCB.

(16) $$\tau_{u}=\sqrt{f_{c}}\left(\frac{23.88}{d_{b}}+0.384\right)$$

(17) $$s_{f}=2G_{f}/\tau_{u}$$

(18) $$G_{f}=\frac{1}{2}\tau_{u}s_{f}$$

where *f_t_* is the tensile strength of concrete, *d* is the SFCB diameter, and *G_f_* is the interface fracture energy. For simplification, the interface is considered fully failed when the relative slip reaches the final slip value of *s_f_* = 10 mm. The bond-slip parameters of the SFCB-concrete interface for the beam specimens are listed in Table 4. The lower G*_f_* of specimen SG7(3) is attributed to three main factors. The increased FRP thickness led to a weakened bond strength. It also resulted in reduced slip values. Additionally, a tendency for splitting failure was promoted.

### 2.2. Unit Geometric Properties and Contact Settings

**Figure 6:**
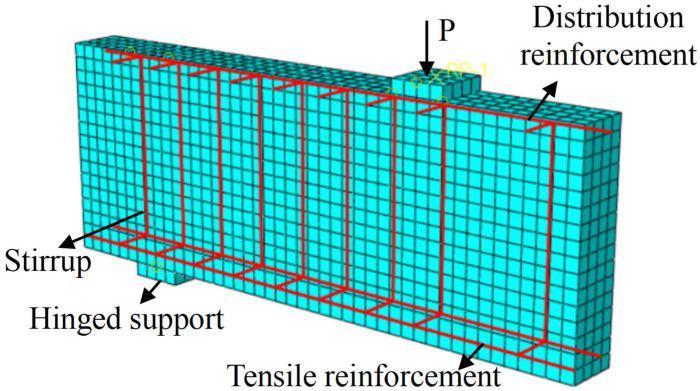
Finite element model and mesh division of the beam.

For different constituent materials of the specimens, appropriate element types should be selected in finite element modeling according to their geometric dimensions and stress characteristics. Specifically, three-dimensional eight-node reduced integration solid elements (C3D8R) were used for concrete and tensile reinforcement; three-dimensional two-node truss elements (T3D2) were employed for compressive steel bars and stirrups; The tensile reinforcement (e.g., steel rebars, GFRP bars, or SFCBs) was modeled using beam elements, with the cross-sectional radius defined according to the specific beam dimensions. Similarly, the bond-slip relationship between the tensile reinforcement and concrete was simulated via connector elements. After meshing, geometric units were constructed between adjacent nodes of the reinforcement and the surrounding concrete. Connectors were assigned using a bushing formulation, with the elastic and plastic parameters of each bushing defined according to the bond-slip parameters provided in Table 4. For compressive steel bars and stirrups, an embedded region approach was used to constrain them within the concrete elements. The geometry and mesh division of the model are shown in Figure 6.

## 3. Model Validation

The above finite element (FE) model was imported into ABAQUS for numerical analysis. The S(3) and SG3 groups were selected as control models. The FE results were extracted and compared with the experimental data to evaluate the accuracy of the numerical simulations.

**Figure 7:**
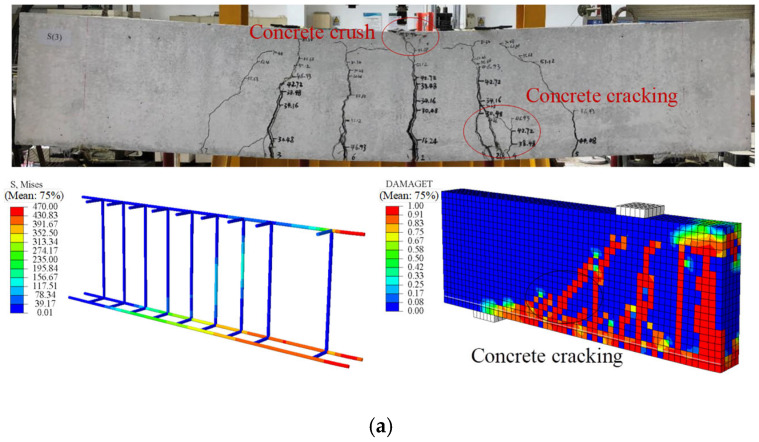

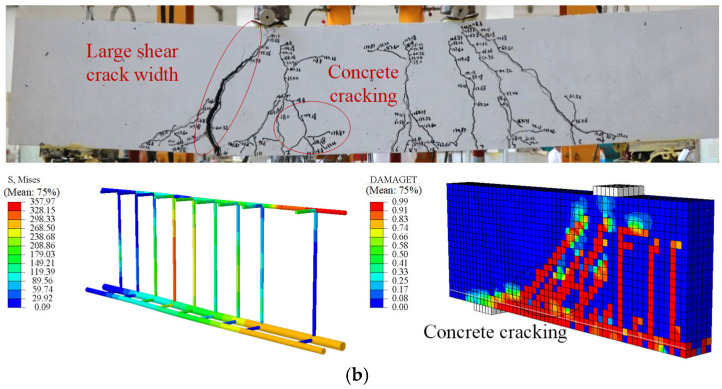
Comparison of failure modes between experiments and finite element calculations: (**a**) S(3); (**b**) SG(3).

**Figure 8:**
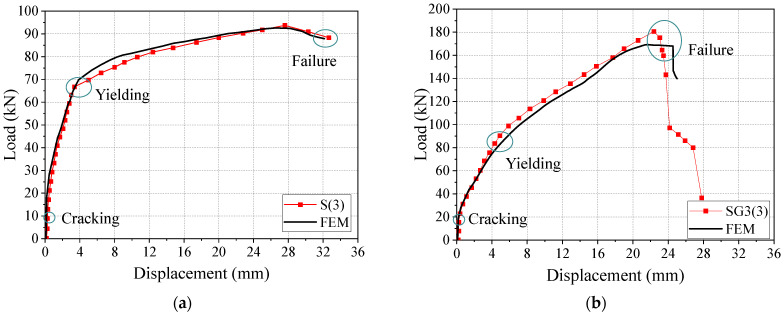
Load–displacement curves: (**a**) S(3); (**b**) SG(**3**).

Figure 7 presents a comparison of the failure modes of the beams between experiments and FE analysis, while Figure 8 shows the load–displacement curves. It can be seen that the failure mode of the reinforced concrete (RC) beams corresponds to a typical flexural failure: the main tensile reinforcement yields, and the concrete in the compression zone is crushed. For beam S(3), the bending process can be divided into two stages: before concrete cracking and from concrete cracking to structural failure, exhibiting a clear bilinear behavior. Comparing FE results with experimental measurements, the ultimate flexural capacity shows an error of 1.24%, and the midspan displacement error is 3.95%.

In specimen SG3(3), flexural and shear capacities were designed to be similar. When the SFCB core steel yielded, the compressive concrete remained intact, with multiple cracks forming in the midspan and bending-shear zones. Failure occurred when concrete in both pure bending and bending-shear regions crushed, although the SFCB had not reached its ultimate tensile strength. Cracks propagated to the beam top, indicating a flexure-shear failure-consistent with experimental observations. The load–displacement curve for beam SG3(3) exhibits a three-stage linear pattern with two clear inflection points. Initially, the uncracked concrete and SFCB work together, resulting in a linear and rapid increase in load. As the load increases, cracks propagate quickly, and the tensile concrete cracks, ceasing to carry load, so the SFCB bears the full load, corresponding to the first inflection point. When the SFCB load reaches the yield of the core steel, the core steel yields, and additional load is transferred to the SFCB fibers, producing the second inflection point. The ultimate load is reached when the SFCB attains its tensile strength (outer fiber fracture) or the compression-zone concrete crushes, marking beam failure.

Thus, SFCB-steel-concrete beams experience three stages under flexural loading: (1) concrete cracking, (2) SFCB core steel yielding, and (3) compression-zone concrete crushing. After core steel yielding, the beam still retains substantial load-bearing capacity, offering a clear advantage over conventional RC or steel/FRP-reinforced beams, aligning with structural safety principles. Comparison between FE predictions and experiments for SG3(3) shows an ultimate flexural capacity error of 6.82% and a mid-span displacement error of 7.29%. Overall, the FE results agree well with the experimental data, demonstrating that the proposed FE model effectively simulates the beam’s flexural behavior.

## 4. Parameter Analysis

**Figure 9:**
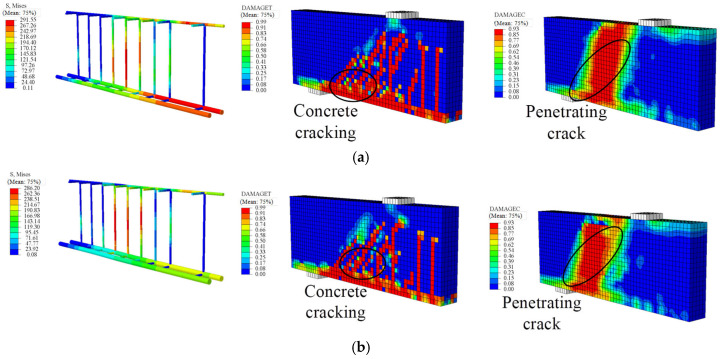
Failure modes of the SG5(3) and SG7(3) beams: (**a**) SG5(3); (**b**) SG7(3).

To investigate the effect of SFCB fiber thickness on the flexural performance of SFCB-RC beams, finite element models were established for beams with fiber thicknesses of 3, 5, and 7 mm, while keeping the core steel unchanged at 10 mm. Figure 9 shows that SG3(3) exhibited flexure-shear failure. For specimens SG5(3) and SG7(3), when the SFCB core steel yielded the compression-zone concrete remained intact, and multiple cracks appeared at mid-span and in the bending-shear regions. When the concrete in the bending-shear zone was crushed, the mid span compression-zone concrete remained uncrushed, and the SFCB had not yet reached its ultimate tensile strength; cracks extended through the beam top at mid-span and in the bending shear zones, indicating shear-dominated failure.

### 4.1. Load–Displacement Curves

**Figure 10:**
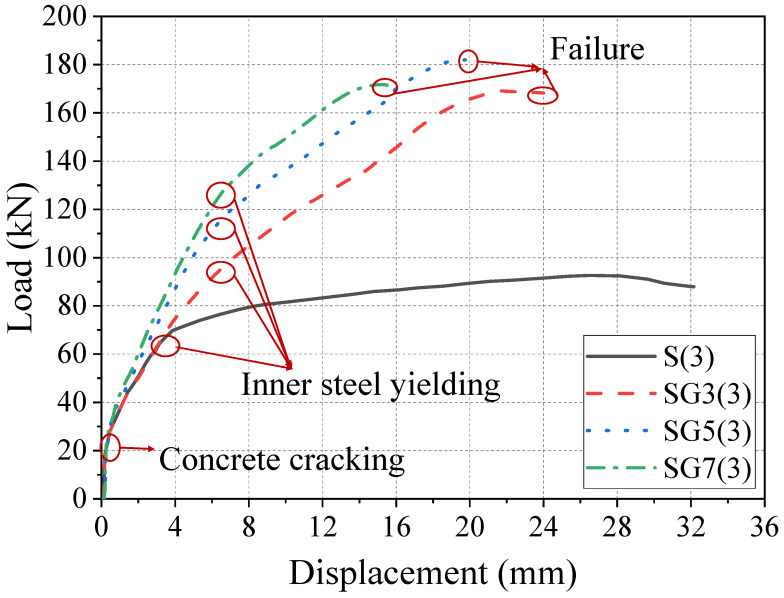
Load–displacement curves of the finite element calculation of reinforced concrete beams.

Figure 10 shows the load–displacement curves from finite element analysis for all reinforced concrete beams. Table 5 lists the key parameters of the beams, where *P_c_*, *P_y_*, and *P_u_* represent the cracking load, yield load, and ultimate load, respectively, and Δ*_c_*, Δ*_y_*, and Δ*_u_* denote the corresponding cracking, yield, and ultimate displacements. Additionally, *μ* represents the ductility index of the beams. The differences between the load–displacement curves of RC beams and SFCB-RC beams were discussed in the previous section and will not be repeated here. From Figure 10, the influence of fiber volume fraction on the load–displacement behavior of SFCB reinforced concrete beams can be observed. After the yielding of steel/core steel, the curves of all beams show clear stage distinctions, indicating that with increasing fiber volume fraction, the strain growth rate decreases under the same steel reinforcement. At the same load level, the RC beam exhibits the largest strain, while beam SG7(3) shows the highest strain among SFCB-reinforced beams.

### 4.2. Cracking Load

**Figure 11:**
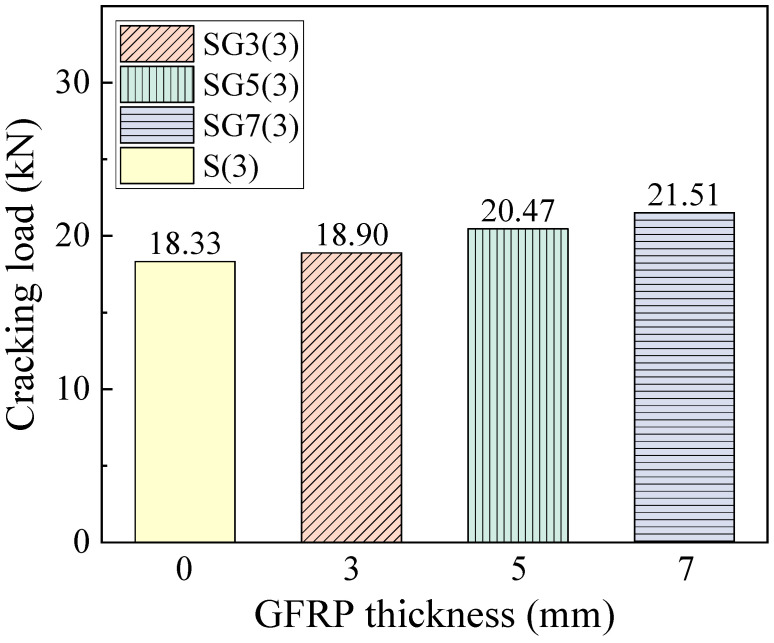
The cracking load of the beams.

The cracking load corresponds to the load at which the concrete in the tension zone reaches its ultimate tensile stress and cracks. Figure 11 presents the cracking loads of the beams. It can be seen that the cracking load increases with fiber thickness. As the fiber thickness increases from 3 mm to 7 mm, the cracking load rises from 18.33 kN to 21.51 kN. Under a constant steel reinforcement ratio, increasing the fiber layer thickness raises the effective reinforcement ratio of the beam, thereby increasing its overall stiffness. A stiffer beam can sustain a higher load before the tension-zone concrete reaches its ultimate tensile stress.

### 4.3. Yielding Load

**Figure 12:**
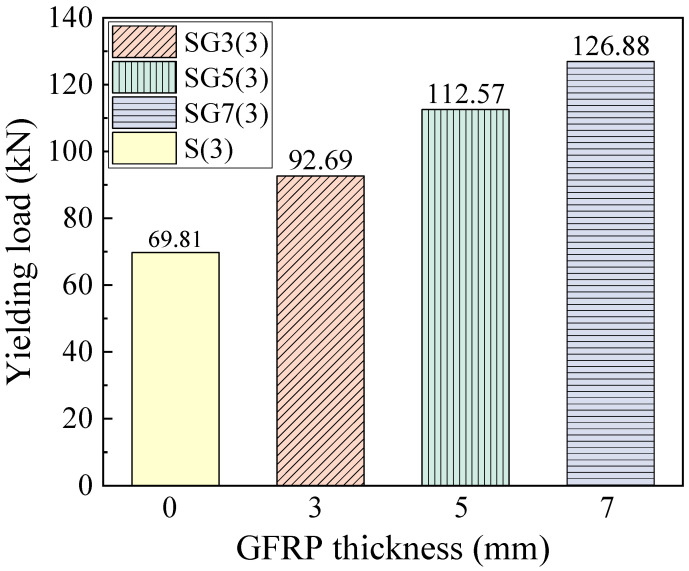
The yielding load of the beams.

The yield load corresponds to the load at which the reinforcement in the beam yields. From the load–displacement curves in Figure 10, SFCB-reinforced beams do not show a flat yielding plateau but instead exhibit a distinct yield point. Using both the curve shapes and finite element data, the corresponding yield loads were determined. Figure 12 presents the yield loads of SFCB-reinforced beams with different fiber thicknesses. It can be seen that the yield load increases with fiber thickness. With a constant steel reinforcement ratio, increasing the fiber thickness raises the beam’s effective reinforcement ratio, thereby increasing its stiffness. Consequently, when the inner core steel bars of the SFCB in the beam reach their yield point, beams with greater stiffness correspond to higher loads.

### 4.4. Ultimate Load

**Figure 13:**
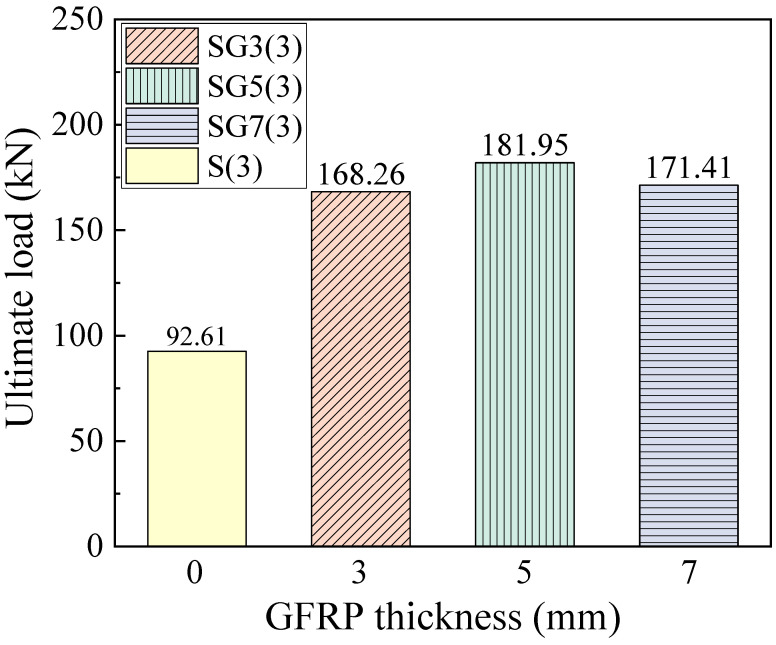
The ultimate load of the beams.

The ultimate load is defined as the point at which either the tensile reinforcement fractures or the concrete in the compression zone crushes, accompanied by a sudden drop in the beam’s load-carrying capacity, indicating beam failure and the attainment of its ultimate strength. Figure 13 presents the ultimate loads of the beams. For SFCB-RC beams with fiber thicknesses of 3, 5, and 7 mm, the ultimate loads are 168.26, 181.95, and 171.41 kN, respectively. The ultimate load of the SG5(3) beam is higher than that of SG3(3) because the increased equivalent reinforcement ratio enhances the ultimate load capacity of the SFCB-RC beam. However, for the SG7(3) beam, when the concrete in the compression zone crushes, the embedded SFCB longitudinal bars have not reached their ultimate tensile strength. As a result, the beam fails before attaining its full flexural capacity, causing the ultimate flexural load of SG7(3) to be lower than that of SG5(3).

### 4.5. Ductility Analysis

**Figure 14:**
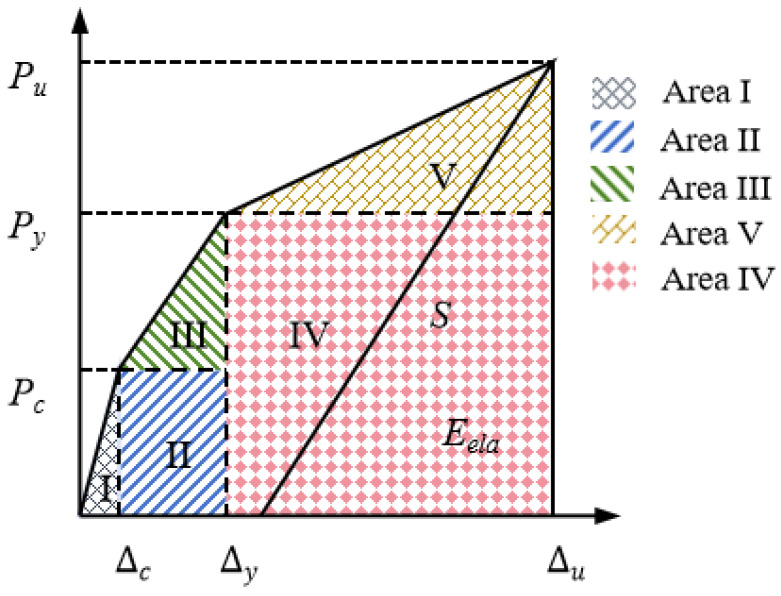
Schematic diagram of the load–displacement relationship of the beam.

Ductility refers to the deformation capacity of a structure from yielding to the maximum load, or during the period after the maximum load before significant strength degradation occurs. The ductility coefficient for S(3) was calculated using *μ* = Δ*_u_*/Δ*_y_*. In this study, a ductility model correlating deformation capacity and energy dissipation is used to evaluate the ductility of SFCB-RC beams [50]. Figure 14 shows the trilinear load displacement response of the beams. The total energy, *E_tot_*, is calculated as the sum of the areas under five regions of the load–displacement curve. The elastic energy, *E_ela_*, is calculated using:

(19) $$E_{\mathrm{ela}}=\frac{P_{u}^{2}}{2S}$$

The unloading branch slope *S* can be simply assumed to be similar to the slope of the curve connecting the ultimate load to the yield load, as this formula has been shown to provide an upper bound for the elastic energy released at failure [51]. Therefore, *S* is calculated using the following equation:

(20) $$S=\frac{P_{y}-P_{c}}{\Delta_{y}-\Delta_{c}}$$

The ductility index, *μ*, is then defined as:

(21) $$\mu =\frac{E_{tot}}{E_{ela}}=\frac{S\left[P_{y}(\Delta_{u}-\Delta_{c})+P_{u}(\Delta_{u}-\Delta_{y})+P_{c}\Delta_{y}\right]}{P_{u}^{2}}$$

**Figure 15:**
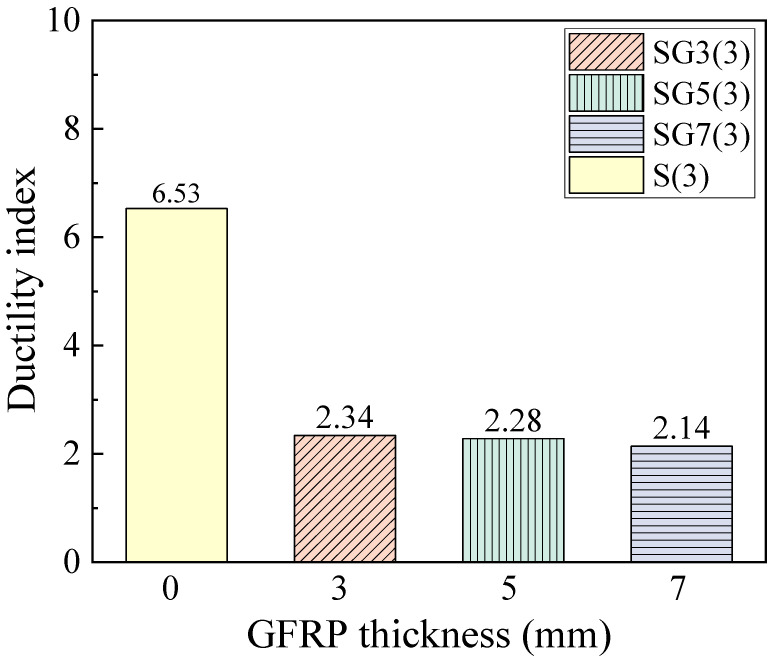
Ductility index of beams.

Figure 15 presents the ductility indices. It is observed that well-reinforced RC beams exhibit the highest ductility. For SFCB-RC beams, the ductility decreases as fiber thickness increases. This is because the elastic energy is primarily provided by the core steel and fiber layer before yielding, while the plastic energy is supplied by the core steel after yielding [31]. Thus, when the core steel is fixed, increasing the fiber thickness does not enhance the plastic deformation capacity but increases the elastic energy, leading to a reduced ductility of the beam.

The decrease in ductility directly reduces the component’s energy dissipation capacity through the formation of plastic hinges. It also diminishes the deformation warning before failure. More critically, it significantly compromises post-earthquake repairability. However, as shown in Figure 10, the increase in FRP thickness raises the ratio of the beam’s ultimate load capacity to its yield load capacity. This indicates an enhanced strength safety margin for the component. A beneficial implication for seismic design is that this provides a greater overstrength basis for structures. It facilitates the implementation of the capacity design principle, such as ensuring a “strong column-weak beam” mechanism. Consequently, the collapse resistance of the structure under severe earthquakes is improved.

The energy-based model used for calculating the ductility index, although widely employed in numerous studies for the comparative analysis of FRP and related composite-reinforced concrete beams and also adopted in this research, still requires further validation through future experiments regarding its general applicability to SFCB-reinforced beams. Developing a more precise evaluation standard for the ductility of SFCB components is an important direction for subsequent research.

## 5. Comprehensive Discussion

This study provides a systematic numerical investigation into the flexural behavior of SFCB-RC beams, with a particular emphasis on the critical yet often overlooked bond-slip effect. The primary findings regarding the influence of outer FRP thickness reveal a significant trade-off between strength and ductility. While increasing the FRP layer enhances the beam’s stiffness, leading to higher cracking and yield loads, it also precipitates an undesirable shift in failure mode from flexural-dominated to shear-dominated for the SG7(3) beam. This indicates that merely increasing the FRP content does not guarantee a proportional improvement in flexural capacity; instead, it may lead to premature concrete crushing before the SFCB’s tensile strength is fully utilized, thereby compromising the structural ductility and safety reserves. The incorporation of the bond-slip model was crucial in capturing these nuanced failure mechanisms accurately, which would have been oversimplified in models assuming a perfect bond.

It can be observed that while several studies have experimentally explored the flexural performance of SFCB-RC beams [16,17,19], high-fidelity finite element analyses that explicitly model the SFCB-concrete interface are scarce. The present study addresses this research need by developing and validating a sophisticated FE model against experimental data, thereby delivering a clear, parameterized understanding of how the FRP thickness governs the structural performance. This work provides valuable theoretical basis and a reliable numerical tool for optimizing the design of SFCB-reinforced concrete structures in future engineering applications.

Some limitations should be noted. The bilinear bond-slip model, though effective under monotonic loading, may not fully represent behavior under cyclic loading, long-term loading, or harsh environments. Thus, the model could overestimate bond performance under those conditions. Future studies should incorporate more advanced models accounting for damage accumulation and environmental effects. Long-term durability factors, such as steel corrosion, alkali attack on GFRP, and creep, were also not considered. Therefore, the findings are primarily applicable to short-term static loading in benign conditions. Further aging tests and long-term simulations are recommended.

Current design codes lack specific guidelines for SFCB-reinforced members. The findings on failure mode transition and the strength-ductility trade-off with increasing FRP thickness can inform future code revisions. A logical next step is to compare these FE results with code predictions for conventional reinforcement and propose appropriate modification factors for practical design.

## 6. Conclusions

Based on the three-dimensional finite element (FE) method, incorporating the bond–slip constitutive model between SFCB and concrete, an FE model of SFCB–steel reinforced concrete beams under flexural loading was established. The influence of SFCB outer fiber thickness (0 mm, 3 mm, 5 mm, and 7 mm) on the flexural capacity of SFCB–steel reinforced concrete beams was investigated, leading to the following conclusions:

1. The reliability of the FE model was validated by comparison with experimental results. The FE model effectively reproduced the flexural behavior of beams, with the failure modes consistent with experimental observations and a high degree of agreement in load–displacement responses. For beam SG3(3), the error in ultimate flexural capacity was 6.82%, while the mid-span displacement error was only 7.29%.
2. The flexural response of SFCB-steel reinforced concrete beams comprises three stages: (i) concrete cracking, (ii) yielding of the inner core steel bars of the SFCB, and (iii) crushing of the concrete in the compression zone. Consequently, SFCB-steel reinforced concrete beams retain significant load-carrying capacity after yielding of the inner steel core, offering clear advantages over conventional steel-or FRP-reinforced beams and aligning more effectively with the safety philosophy of structural design.
3. Both cracking load and yield load increase with the thickness of the SFCB outer fiber layer, owing to the increase in the equivalent reinforcement ratio *ρ_hE_*, which enhances the overall stiffness of the beam. As a result, the beam sustains higher loads before the tensile-zone concrete reaches its ultimate tensile stress, and the corresponding load at steel core yielding increases with beam stiffness.
4. The ultimate load of beam SG5(3) was greater than that of SG3(3), as the increased equivalent reinforcement ratio enhanced the flexural capacity of the SFCB-concrete beam. However, excessive fiber thickness (7 mm) led to shear failure, where the compression-zone concrete was crushed before the SFCB reached its ultimate tensile strength, thereby reducing the load-bearing capacity.
5. Among all specimens, the adequately reinforced steel-concrete beam exhibited the best ductility. For SFCB-concrete beams, the ductility index decreased with increasing fiber thickness. This is because when the inner core steel reinforcement remains constant, increasing the fiber thickness enhances the elastic energy capacity of the beam without improving its plastic energy capacity, thus leading to reduced ductility.

## Figures and Tables

**Table 1:** Details of beams.

Specimen	*A_s_* (mm^2^)	*A_f_* (mm^2^)	*ρ_s_* (%)	*ρ_f_* (%)	*ρ_h_* (%)	*ρ_hE_* (%)
S(3)	235.50	0.00	0.32	0.00	0.32	0.32
SG3(3)	235.50	367.38	0.32	0.50	0.82	0.44
SG5(3)	235.50	706.50	0.32	0.97	1.29	0.56
SG7(3)	235.50	1120.98	0.32	1.54	1.87	0.70

**Table 2:** Parameters of the concrete plastic damage model.

Angle of Dilatancy	Flow Potential Offset	Ultimate Strength Ratio of Biaxial Compression to Uniaxial Compression	Invariant Stress Ratio	Viscosity Coefficient
30	0.1	1.16	0.667	0.0005

**Table 3:** Material performance parameters of bars.

Specimen	*t_f_* (mm)	*D* (mm)	*E_I_* (GPa)	*E_II_* (GPa)	*f_y_* (MPa)	*f_sfy_* (MPa)	*f_fu_* (MPa)	*f_su_* (MPa)	*f_sfu_* (MPa)
Steel	0	10	206	/	470	/	/	557	/
GFRP	/	12	50.7	/	/	/	901	/	/
SG3	3	16	100	26.8	/	222	/	/	760
SG5	5	20	82.3	33.9	/	190	/	/	811
SG7	7	24	72.6	35.6	/	155	/	/	821

**Table 4:** SFCB-concrete bonding interface parameters.

Specimen	*τ_u_* (MPa)	*s_u_* (mm)	*G_f_* (MPa·mm)
S(3)	11.0	2.70	55.00
SG3(3)	9.70	3.60	48.50
SG5(3)	8.16	3.60	40.80
SG7(3)	7.13	3.60	35.65

**Table 5:** Finite element calculation results of reinforced concrete beams.

Specimen	*P_c_* (kN)	*P_y_* (kN)	*P_u_* (kN)	Δ*_c_* (mm)	Δ*_y_* (mm)	Δ*_u_* (mm)	*μ*
S(3)	18.33	69.81	92.61	0.18	3.86	26.50	6.53
SG3(3)	18.90	92.69	168.26	0.21	6.14	24.00	2.34
SG5(3)	20.47	112.57	181.95	0.32	6.16	19.75	2.28
SG7(3)	21.51	126.88	171.41	0.25	6.54	15.95	2.14

## Data Availability

The data that support the findings of this study are not publicly available due to privacy restrictions but are available from the corresponding author upon reasonable request.
